# Effect of Ultimate pH on Postmortem Myofibrillar Protein Degradation and Meat Quality Characteristics of Chinese Yellow Crossbreed Cattle

**DOI:** 10.1155/2014/174253

**Published:** 2014-08-13

**Authors:** Peng Li, Tiantian Wang, Yanwei Mao, Yimin Zhang, Lebao Niu, Rongrong Liang, Lixian Zhu, Xin Luo

**Affiliations:** ^1^Department of Food Science and Engineering, Shandong Agricultural University, Tai'an, Shandong 271018, China; ^2^Department of Food Science and Engineering, Qingdao Agricultural University, Qingdao, Shandong 266109, China

## Abstract

This paper describes the complex effects of postmortem ultimate pH (pHu) on Chinese Yellow crossbreed cattle quality during postmortem ageing and provides an explanation of how pHu affects beef tenderness. High pHu beef had the highest initial tenderness (*P* < 0.05) compared with other groups at 1 day postmortem. Intermediate and low pHu beef had similar initial WBSF at 1 day postmortem, but intermediate pHu beef had slower tenderization rate than low pHu beef (*P* < 0.05). Purge loss, cooking loss, L*, a*, and b* values decreased with increasing pHu during ageing (*P* < 0.05). Myofibril fragmentation index (MFI) was higher in high pHu beef than intermediate and low pHu beef throughout ageing (*P* < 0.05). Protein degradation studies found that desmin and troponin-T appeared degraded within 0.5 h postmortem for high and low pHu beef, compared to >2 days for intermediate pHu beef. Overall, Chinese Yellow crossbred cattle tenderness is related to pHu, which may be affected by proteolytic enzymatic activity. Therefore, pHu may be used to predict beef tenderness and other quality characteristics during postmortem ageing. To achieve consistent tenderness, different ageing times should be used, depending on pHu.

## 1. Introduction

Tenderness has been identified as one of the most important characteristics that determine the consumer eating satisfaction of beef [1]. Variability in the meat tenderness is the most critical quality problem facing the beef industry. Variable tenderness is a limiting factor for product acceptability, causing a reduction in beef consumption. Consumers are concerned with uniform in the tenderness of beef and would probably pay a higher price for beef as long as it has satisfactory tenderness [2].

Tenderness and rate of tenderization depend on many intrinsic (species, animal age, type of muscle, and muscle location) and extrinsic factors (preslaughter stress, slaughter conditions, and postslaughter handling) of the animal and on their interaction. Beef tenderness has long been associated with pHu, with meat that achieves high pHu (>6.2) and low pHu (<5.8) being acceptably tender after appropriate ageing time [3]. Ultimate pH (pHu) can also markedly affect other meat quality parameters, including colour, water-holding capacity, and shelf life. Thus, pHu has been widely used as an indicator of potential meat quality [1, 4]. Additionally, the rate of tenderization is related with pHu; high pHu meat tenderises more rapidly than low pHu meat during ageing [5, 6].

pHu variation is often associated with postmortem muscle glycogen content and metabolism, which is affected by many factors including psychological stress, animal diet, season, transport, and lairage times, thus affecting initial meat shear force values and rate of tenderization [7]. Although many factors influencing pHu values have been discussed, no single factor can explain more than 50% of the variation in pHu. Thus, the inconsistencies in meat quality due to variation in pHu will continue to pose a problem to meat processors until the exact relationship between all the factors is understood.

Meat tenderness may be influenced by the breakdown of myofibrillar structure protein due to postmortem proteolytic activity. Much research has been focused on determining the contribution of various myofibrillar proteins to meat tenderness. It has been consistently reported that tender meat has faster and more extensive degradation of desmin, troponin-T, nebulin, and titin, compared with tough meat. By monitoring the degradation of these proteins during ageing, researchers have attempted to determine a suitable ageing time to obtain acceptable tenderness [8, 9].

At present, little information is available concerning the effects of pHu on beef quality and myofibrillar protein degradation in Chinese cattle. Yellow Cattle is the most common Chinese breed, accounting for approximately 80% of the national herd and crossbreeds predominate in commercial trade [10]. The inconsistent tenderness of beef is a severe problem in Chinese Yellow cattle, resulting in greater uncertainty in producing consistent high-value chilled beef. Tenderness of meat from Chinese Yellow crossbred cattle may be improved by extending ageing time [10], but much energy and time may be wasted if all carcasses are given extended ageing, when some carcasses may have good tenderness without extended ageing.

This paper describes the complex effects of pHu on beef quality characteristics from Chinese Yellow crossbreed cattle during postmortem aging and provides an explanation of how pHu affects beef tenderness and rate of tenderisation.

## 2. Materials and Methods

### 2.1. Animals, Experimental Design, and Treatments

Thirty Chinese Yellow crossbreed cattle [Luxi (Chinese native Yellow cattle) × Limousine; Limousine has a lower input than the crossbreed, about 24 months old and of mean live weight 450.4 ± 48.3 kg (mean ± SD)] were selected from a commercial feedlot (Shandong Xinlv Food Ltd., China). The cattle were kept in lairage overnight and stunned by electrical stunning prior to slaughter. The carcasses were transferred to a chilling room (2 ± 2°C) within 30 min postmortem. About 10 g of Longissimus dorsi (LD) muscle samples was cut from the same side of each carcass at 0.5, 3, 6, 12, and 24 h postmortem, snap-frozen in liquid nitrogen, and stored at −80°C until analysed. At 24 h postmortem, the pHu of LD was measured using a portable pH meter (SenvenGo, Mettler Toledo, Switzerland) and LD was removed from each carcass. Based on pHu, the muscles were segregated into three groups: high (pH > 6.2, *n* = 4), intermediate (pH 5.8–6.2, *n* = 7), and low (pH < 5.8, *n* = 19) pHu beef. Each of the LD muscles was cut into five segments, vacuum-packaged in polyethylene bags, weighted, and continuously stored in incubators with air temperature of 4 ± 1°C. Then, all the samples were stored for 1, 3, 5, 7, and 9 d postmortem.

### 2.2. Sampling and Measurements

*Purge Loss (PL)*. At 24 h postmortem, samples were weighed and then vacuum packaged. Purge loss during vacuum storage was determined by weighing samples after storage. Before weighing the samples, they were dried with paper towels. Purge loss was expressed as percentage of weight loss.

*Color Measurement*. After purge loss measurement, samples were exposed in the air for 30 min before measurement. Meat color of the samples was measured using a colorimeter (SP62, X-Rite Inc., Gryndville, MI, USA) with an 8 mm diameter measuring aperture, illuminant D65, and CIE L*  a*  b* color scale. Color coordinate values were recorded as L* lightness, a* redness, and b* yellowness values.

*Cooking Loss (CL)*. The samples after measurements of PL and color were weighed, placed individually in plastic bags, and immersed in a water bath at 80°C until they reached an internal temperature of 75°C. The temperature was monitored using thermocouples inserted in the center of the samples. After cooking, the samples were chilled at room temperature and then stored in a refrigerator overnight, surface dried, and weighed again. CL was determined by expressing cooked sample weight as a percentage of precooked samples weight.

*Tenderness*. After measurements of CL, the same muscles were used for the determination of Warner-Bratzler shear force (WBSF). Shear force measurements were performed as described previously by Luo et al. [11]. Six cores (1.25 cm diameter) were excised, parallel to the longitudinal orientation of the muscle fibers. The cores were sheared once using a texture analysis machine (TA-XT2i Stable Micro System, Godalming, England) with a HDP/BSW blade. The average of readings for the cores (1.25 cm diameter) from the same sample was the WBSF value (kg).

*Myofibril Fragmentation Index*. Myofibril fragmentation index (MFI) was determined using a slightly modified version of the procedure of Culler et al. [12]. Small samples were taken from muscles at 1, 3, 5, 7, and 9 d postmortem, immediately frozen in liquid nitrogen, and stored at −80°C until analysis. The frozen samples were minced in a cutter, after all visible fat and connective tissue had been removed. Four grams of minced meat was homogenized for 30 s in 40 mL of 0.02 M potassium phosphate buffer (pH 7.0) containing 100 mM KCl, 1 mM EGTA, 1 mM MgCl_2,_ and 1 mM NaN_3_ at about 4°C using a mixer. After centrifugation at 1000 ×g for 15 min the supernatant was discarded. The sediment was resuspended in 40 mL buffer, stirred, and centrifuged and the supernatant discarded. The sediment was resuspended in 10 mL buffer and filtered through a polyethylene strainer to remove connective tissue and fat. An additional 10 mL of the buffer was used to facilitate the passage of myofibrils through the strainer. Determination of the protein concentration of the suspension was done by the biuret method [13]. Then, the suspension was diluted with buffer to 0.5 ± 0.05 mg/mL protein concentration. MFI is the value of absorbance of the myofibrillar suspension, measured at 540 nm multiplied by 200.

*Gel Electrophoresis and Western Blotting*. The myofibrillar protein fraction was separated using a modification of the method described by Sikes et al. [14]. Four-gram sample from each muscle stored at −80°C was knife-minced and homogenized with 40 mL of extraction buffer (50 mM Tris-HCl, pH 7.0, 100 mM KCl, and 5 mM EDTA) for 2 min. The sample was then centrifuged at 1000 ×g for 15 min at 4°C, the supernatant was discarded and the pellet was resuspended in 20 mL of extraction buffer. The pellet was washed a further four times by suspending in 20 mL of extraction buffer and sedimenting at 1000 ×g for 15 min at 4°C. Lastly, the pellet (myofibrillar protein fraction) was resuspended in 20 mL of extraction buffer. Protein concentration of the myofibrillar protein faction was determined by using the biuret method. The samples were diluted to 2 mg/mL in tracking dye (62.5 mM Tris-HCl (pH 6.8), 10% glycerol, 2% SDS, 5% 2-mercaptoethanol, and 0.02% bromophenol blue) in preparation for SDS-PAGE. All samples were immediately denatured in a water bath at 95°C for 5 min, cooled, and then stored at −20°C until further analysis.

SDS-PAGE gel electrophoresis and western blotting were performed as previously described [8, 9] with some modifications. Denatured myofibril protein (40 *μ*g) samples were loaded on 12.5% SDS-polyacrylamide resolving gels with an acrylamide: bisacrylamide weight ratio of 37.5 : 1. The gel was run in 25 mM Tris-HCl containing 192 mM glycine, 1 mM EDTA, and 1% SDS at a constant current of 20 mA for 6 h. Prestained Multicolor Broad Range Protein Marker (Tiangen, Beijing, China; 245 kDa to 11 kDa) was used as the molecular weight marker of SDS-PAGE. After SDS-PAGE, gels were equilibrated for 30 min at room temperature in transfer buffer (25 mM Tris, 192 mM glycine, and 15% vol/vol methanol).

Following SDS-PAGE, proteins were transferred onto Immobilon-P PVDF membranes (Millipore, IPVH00010) and blocked by incubating the membrane with 5% nonfat dry milk powder diluted with PBST (0.08 M Na_2_HPO_4_, 0.02 M NaH_2_PO_4_, 0.1 M NaCl, and 0.1% Tween) overnight at 4°C. Membranes were then washed three times with PBST and incubated with the chosen primary antibody for 1 h at room temperature. Primary (monoclonal) antibodies from mouse, including antibodies to desmin (Sigma, D1033) and troponin-T (Sigma, T6277), were diluted to 1 : 1000 in PBST. After incubation with primary antibody, membranes were washed three times with PBST for 10 min each time. Subsequently, membranes were incubated with the secondary antibody (1 : 4000, goat anti-mouse IgG alkaline phosphatase-conjugated secondary antibody, Sigma, A3562) in PBST for 2 h at room temperature, and the membrane was washed three times with PBST for 10 min each time. Alkaline phosphatase staining was performed with BCIP/NBT Alkaline Phosphatase Colour Development Kit (Beyotime) according to manufacturer's instructions.

### 2.3. Statistical Analysis

The effects of pHu and ageing time on WBSF and MFI were evaluated using the analysis of variance. The mathematical model for PL, CL, L*, a*, and b* included fixed effect due to pHu, ageing time, and pHu × ageing. When significant, differences between means were tested by the least significant difference (LSD) procedure. Statistical significance was set at *P* < 0.05. All figures were plotted using SigmaPlot software (version 12.0, Systat, San Jose, CA, USA). Statistical analyses were performed with SPSS 19.0 software (SPSS Science, Chicago, IL, USA).

## 3. Results and Discussion

### 3.1. Tenderness

**Figure 1:**
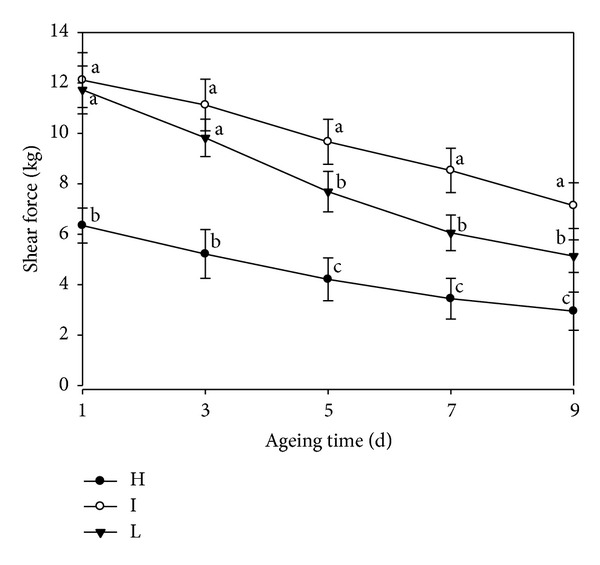
Effect of ultimate pH (pHu) and ageing time on shear force value of M. Longissimus dorsi from Chinese Yellow crossbreed cattle, stored at 4°C. Table values are pHu group means ± standard deviation (SD). (H = high pHu group, meat pH > 6.2; I = intermediate pHu group, pH 5.8–6.2; L = low pHu group, pH < 5.8). Different letters at the same ageing time indicate significant differences (*P* < 0.05).

The Warner-Bratzler shear force (WBSF) changes caused by the effects of pHu and ageing time (during 9 days postmortem) are presented in Figure 1, which showed that tenderness and rate of tenderization were related with pHu. pHu and ageing time both affected (*P* < 0.05) the WBSF of LD. High pHu beef was lower WBSF, achieving good tenderness [15] at 1 day postmortem and remained more tender (*P* < 0.05) than low and intermediate pHu meat during the ageing period. Results showed that the WBSF values were not different (*P* ≥ 0.05) between low and intermediate pHu meat at 1 day postmortem. But during ageing period, the WBSF of intermediate pHu meat decreased more slowly than low pHu meat (*P* < 0.05).

Our findings are similar to previous research that has demonstrated that high pHu meat is consistently more tender than low and intermediate pHu meat even after extended ageing time and that tenderisation was delayed in meat with intermediate pHu [4, 16]. But with regard to ageing time, our results differ from some other results [17], possibly due to different cattle breeds and ageing conditions among the studies.

### 3.2. Myofibrillar Fragmentation Index

**Figure 2:**
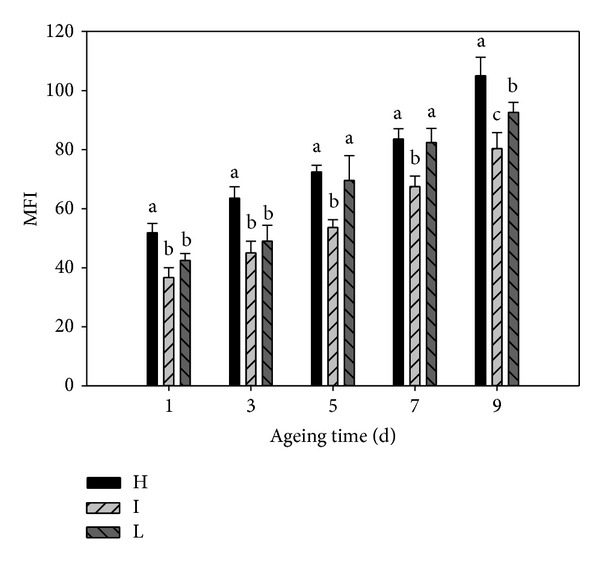
Effect of pHu and ageing time on myofibril fragmentation index of M. Longissimus dorsi samples from Chinese Yellow crossbreed cattle stored at 4°C. (H = high pHu group, meat pH > 6.2; I = intermediate pHu group, pH 5.8–6.2; L = low pHu group, pH < 5.8). Different letters at the same ageing time indicate significant differences (*P* < 0.05).

The relationship between pHu and MFI is shown in Figure 2. MFI significantly increased among the three pHu groups from 1 to 9 d postmortem (*P* < 0.05). During ageing, high pHu group had the highest MFI (*P* < 0.05) compared with the low and intermediate pHu groups. The intermediate pHu group had the lowest MFI and the differences were significant (*P* < 0.05) from day 5 compared with the low pHu group.

In general, meat tenderization is mainly due to ultrastructural changes that weaken the integrity of the myofibers in the muscle tissue [18]. The MFI has been shown to be a good indicator of the extent of muscle myofibrillar protein degradation under postmortem conditions, and the MFI increase is the result of rupture of myofibrils in the I-band of the sarcomere during postmortem storage [19]. Many studies indicate that MFI is related to meat pH and ageing time at a specified time postmortem, which is consistent with meat tenderisation. MFI is strongly correlated with WBSF and postmortem sensory tenderness [7]. Our measurements revealed that at 24 h postmortem high pHu group had a higher postmortem proteolytic activity compared to others [20]. To some extent, these results may explain why the beef from the high pHu group had better initial tenderness.

### 3.3. Water Holding Capacity

The effects of pHu and ageing time on purge loss and cooking loss of LD muscle is presented in Table 1. Both pHu and ageing time significantly affected the purge loss (*P* < 0.001). Purge loss of all the pHu groups increased as ageing time was extended (*P* < 0.05). Purge loss was significantly lower for the high pHu samples than for the other two groups (*P* < 0.05). During the ageing period, the trend for intermediate pHu beef was in between those groups. This result is in agreement with the results of previous reports, showing that the extent of the pH decrease is a key factor influencing meat quality [21]. Moreover, less cooking loss was observed in the high pHu group than the intermediate and low pHu beef (*P* < 0.05) at all ageing periods. All groups had no difference in percentage of cooking loss throughout 9 days of postmortem ageing (*P* ≥ 0.05). This result is similar to previous reports [22], which stated that muscle pH affected cooking loss and was not affected by ageing time.

### 3.4. Meat Color

Each colour variable was affected by the pHu group and ageing time (*P* < 0.001). An interaction (*P* < 0.001) was also observed between ageing time and pHu after ageing (Table 1). At longer ageing times, L*, a*, and b* increased consistently (*P* < 0.05). The meat from the high pHu group (pH > 6.2) was darker, with lower meat surface L*, a*, and b* values than meat from the other pH groups (*P* < 0.05). There are two explanations in the literature for dark color of high pH meat: (1) due to less light scattering, so that less light is reflected [23], and (2) due to high mitochondrial oxygen consumption in high pH meat, favoring formation of dark deoxymyoglobin [24, 25]. Both mechanisms may be involved in the dark color of meat samples from the high pHu group in this study. Li et al. [10] reported that L* value is correlated with WHC of muscles. Meat with intermediate pHu values seemed to have more rapid linear increase in a* values during storage than samples from the low or high pHu groups. In high pHu meat, a* values were the highest (17.4) at day 3 (*P* < 0.05) but decreased to 12.8 by 9 days ageing.

### 3.5. Degradation of Desmin and Troponin-T in the Three pHu Groups

**Figure 3:**
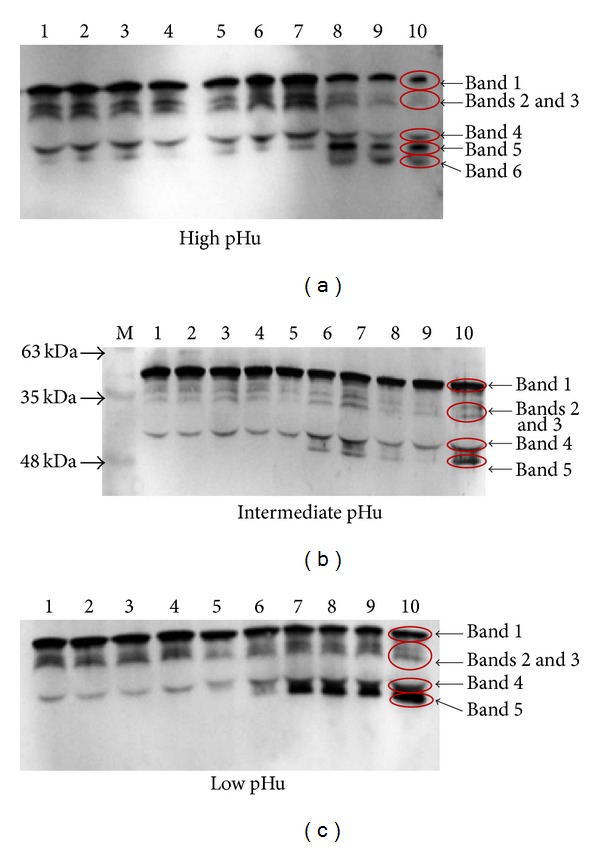
Representative Western blot of desmin and degradation products. Each lane was loaded with 40 *μ*g of protein. M is the molecular weight marker. Lanes 1 to 10 represent the variability in desmin degradation from high, intermediate, and low pHu beef held at 4°C for 0.5 h, 3 h, 6 h, 12 h, 24 h, 2 d, 3 d, 5 d, 7 d, and 9 d postmortem. Band 1 = 54 kDa, Bands 2 and 3 = 50 and 47 kDa, Band 4 = 41 kDa, Band 5 = 39 kDa, and Band 6 = 34 kDa.

Desmin degradation pattern of the beef samples from three different pHu groups in the western blot analysis is shown in Figure 3. From 0.5 h to day 3 postmortem, intact desmin (54 kDa) as well as 50, 47, 41, 39, and 34 kDa degradation products were present in abundance in the high pHu beef. But from day 5 postmortem, intact desmin as well as 50, 47, and 41 kDa degradation products decreased greatly, while a 39 kDa product increased at longer storage times. The degradation pattern of desmin in low pHu beef developed more slowly than in high pHu beef, where a 39 kDa degradation product did not appear until day 3 and no 34 kDa product was observed (Figure 3). Compared to the high or low pHu beef, the intermediate pHu beef degraded much slower, with almost no disappearance of desmin (54 kDa) during ageing (Figure 3).

**Figure 4:**
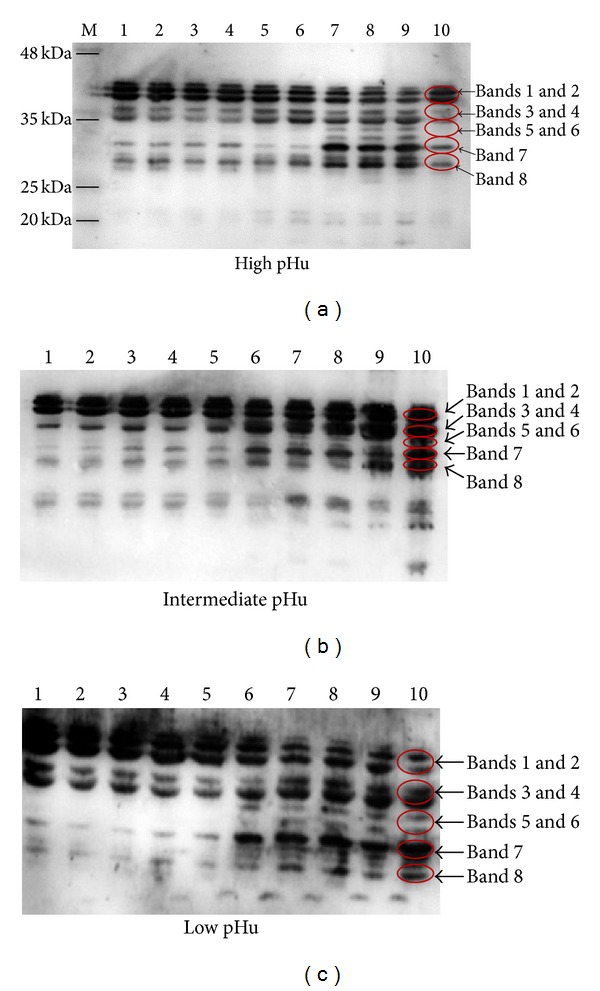
Representative Western blot of troponin-T and degradation products. Each lane was loaded with 40 *μ*g of protein. M is the molecular weight marker. Lanes 1 to 10 represent the variability in troponin-T degradation from high, intermediate, and low pHu beef aged at 4°C for 0.5 h, 3 h, 6 h, 12 h, 24 h, 2 d, 3 d, 5 d, 7 d, and 9 d postmortem. Bands 1 and 2 = 42 and 40 kDa, Bands 3 and 4 = 38 and 36 kDa, Bands 5 and 6 = 34 and 32 kDa, Band 7 = 30 kDa, and Band 8 = 28 kDa.

A representative immunoreactive troponin-T bands blot is shown in Figure 4. Bands 1 and 2 (molecular weight 42 and 40 kDa) likely represented isoforms of intact troponin-T, which is consistent with previous research [26]. The remaining bands (3 and 4, 5 and 6, 7, and 8, molecular weight 38, 36, 34, 32-, 30, and 28 kDa separately) were likely degradation products or a combination of intact isoforms and degradation products. From 0.5 h postmortem, intact troponin-T isoforms of Bands 1 and 2 began decreasing and degradation products Bands 3 and 4 increased during ageing in both high and low pHu beef. Furthermore, in high pHu beef, there was also distinct appearance of degradation product Bands 7 (30 kDa) and 8 (28 kDa) from 0.5 h postmortem, and the intensity increased noticeably from day 3 postmortem, along with Bands 5 and 6 appearing clearly. However, in low pHu beef, intact troponins Bands 1 and 2 were present. Degradation Bands 5, 6, 7, and 8 increased markedly from day 2 postmortem. Among the degradation products, Band 7 (30 kDa) increased markedly during ageing. In contrast to high and low pHu beef, breakdown of intact troponin-T in the intermediate pHu beef was delayed, with breakdown product Bands 3 and 5 and 6 appearing after 3 days postmortem and in smaller quantities (Figure 4).

Desmin is important to the ultrastructure of muscle as it is a constituent of costameres and intermediate filaments that anchor myofibrils sarcolemma and link adjacent myofibrils to each other at the Z-disk level, respectively. The postmortem degradation of desmin has been found to be concomitant with meat tenderization [27]. By determining the difference in the postmortem muscle proteolysis among the three different pHu groups in this study, we found postmortem degradation of desmin was faster in high pHu beef than others, and the intermediate pHu beef had the slowest degradation, indicating that the rate and extent of myofibrillar protein degradation vary with muscle pHu. These results are partly supported by a previous study on postmortem bull LD where desmin disappeared faster in high pHu than in low pHu. In this study, it was readily apparent that there were some different degradation products between high and low pHu beef. In high pHu beef, desmin degradation fragments clearly resulted in the accumulation of a high molecular weight fragment (39 kDa). Furthermore, in low pHu beef desmin was effectively degraded, but no clear electrophoretic fragments appeared, rather, a progressive disappearance of the initial desmin band (54 kDa) and the appearance of a continuous smear of degradation products was observed (Figure 3). An explanation for the observed phenomenon is possibly the differences in calpains and cathepsin B activities at the extremes of postmortem muscle pH.

Troponin-T is well known to be degraded mainly into the approximately 30 kDa products during postmortem ageing. Many researchers have repeatedly shown that a 30 kDa degradation product of troponin-T increases with muscle ageing and is associated with tenderization. For example, Lametsch et al. [18] recently reported that, at 24 h postmortem, the 30 kDa band is present in tender bovine muscle and yet is not detected in tough bovine muscle. Results of this study showed that in high pHu beef the troponin-T degradation process started immediately after slaughter, with the clear appearance of a 30 kDa product. In the low pHu group, the degradation pattern seemed to be similar to that of the high pHu beef. Troponin-T related fragments with 38 and 36 kDa bands were detected within 24 h postmortem. Thereafter, the degradation products increased noticeably with ageing, but the 30 kDa did not become clear until day 2 postmortem. In intermediate pHu beef, troponin-T degradation was much slower, with only very weak degradation visible within 24 h postmortem, and almost no disappearance of intact troponin-T until day 3 postmortem, and decreasing very slowly for the remainder of the storage period. These findings were similar to the results reported by Baron et al. [28], who found that troponin-T breakdown products began to appear between 2 and 3 day postmortem.

Many reports have indicated that postmortem degradation of muscle proteins not only affects meat tenderness [29], but also determines the amount of purge loss during ageing and cooking. Pearce et al. [30] found that a high level of desmin degradation is associated with increasing WHC during postmortem ageing. The present study also found that intermediate pHu (pH 5.8–6.2) was related to limited degradation of desmin and troponin-T, which may explain why the high pHu meat had better WHC. This study also found that the protein degradation intensity was affected by postmortem muscle pHu, which may partly explain why intermediate pHu beef tenderization rate during ageing was slower than for high (>6.2) or low (<5.8) pH groups. These results are consistent with previous research showing that nebulin and titin degradation was slowest at pHu 6.0–6.3 [27], corresponding to the intermediate pH group in this study.

## 4. Conclusion

This study indicates that pHu is an important factor to affect the quality of Chinese Yellow crossbreed cattle, especially for tenderness, including initial tenderness and tenderness changes during the postmortem conversion of muscle to meat. The inconsistency of tenderness among the different pHu groups may be due to the low level of postmortem proteolysis at intermediate pH ranges (5.8–6.2). The rapid tenderisation of high (pH > 6.2) and low pHu beef (pH < 5.8) was likely due to the early postmortem degradation of cytoskeletal proteins such as desmin and troponin-T, possibly due to the immediate activation of *μ*-calpain at high pH, and cathepsins at low pH. Tenderisation of intermediate pHu meat was the slowest, possibly due to limited proteinase activities during the first days of ageing.

## Figures and Tables

**Table 1:** Effect of ultimate pH (pHu) and ageing time on purge loss (PL), cooking loss (CL), and meat color of M. Longissimus dorsi in Chinese Yellow crossbreed cattle (means ± SEM).

	pHu group	Ageing time					SEM	Significance		
		1 d	3 d	5 d	7 d	9 d		pHu	Time	pHu × Time
PL%	H	0.58^cB^	0.64^bC^	0.84^bC^	1.02^bC^	1.67^aC^				
	I	0.88^cB^	1.15^cB^	1.70^bB^	2.09^abB^	2.24^aB^	0.08	∗∗∗	∗∗∗	NS
	L	1.45^cA^	1.83^cA^	2.40^bA^	2.74^bA^	3.31^aA^				

CL%	H	16.05^B^	15.81^B^	17.59^B^	16.83^B^	17.64^B^				
	I	22.68^A^	20.97^A^	23.70^A^	23.63^A^	25.10^A^	5.75	∗∗∗	NS	NS
	L	25.33^A^	22.54^A^	25.73^A^	25.49^A^	27.6^A^				

L∗	H	32.13^cC^	35.63^bC^	37.31^aC^	35.24^bB^	35.43^bC^				
	I	37.49^dB^	39.15^cB^	40.80^bB^	43.38^bA^	44.74^aB^	4.42	∗∗∗	∗∗∗	∗∗∗
	L	41.51^cA^	43.01^bA^	43.40^bA^	43.95^aA^	46.16^aA^				

a∗	H	13.31^cC^	17.39^a^	16.89^abC^	15.93^bC^	12.79^cC^				
	I	14.83^dB^	16.44^c^	16.72^bcB^	17.28^bB^	19.61^aA^	2.87	∗∗∗	∗∗∗	∗∗∗
	L	17.41^A^	16.13^c^	17.68^aA^	17.85^bA^	16.41^bB^				

b∗	H	3.97^bC^	5.42^a^	4.83^aC^	4.65^aB^	3.15^bB^				
	I	5.13^cB^	5.66^c^	6.73^bB^	8.56^aA^	8.44^aA^	1.53	∗∗∗	∗∗∗	∗∗∗
	L	7.35^bA^	5.98^b^	7.60^bA^	8.64^aA^	8.70^aA^				

NS: not significant; ***P < 0.001. a, b, c, d = *P* < 0.05 in rows (ageing time effect). A, B, C = *P* < 0.05 in columns (pHu effect).
